# Engineering Electronic Structure and Band Alignment of 2D Mg(OH)_2_ via Anion Doping for Photocatalytic Applications

**DOI:** 10.3390/ma14102640

**Published:** 2021-05-18

**Authors:** Shunnian Wu, Hasanthi L. Senevirathna, P. Vishakha T. Weerasinghe, Ping Wu

**Affiliations:** Entropic Interface Group, Engineering Product Development, Singapore University of Technology and Design, 8 Somapah Road, Singapore 487372, Singapore; shunnian_wu@sutd.edu.sg (S.W.); hasanthi_senevirathna@mymail.sutd.edu.sg (H.L.S.); puwakdandawe@mymail.sutd.edu.sg (P.V.T.W.)

**Keywords:** 2D Mg(OH)_2_, anion doping, first principles calculation, band gap, band alignment

## Abstract

The wide bandgap of 2D Mg(OH)_2_ inhibits its applications in visible-light photocatalytic applications. Besides, its mismatched band alignment to the redox potential of O_2_/H_2_O, brings about low efficacy of water-splitting performance. Therefore, to release the powder of 2D Mg(OH)_2_ in photocatalytic research, we explore anion doping strategies to engineer its electronic structure. Here, anion doping effects on electronic properties of 2D Mg(OH)_2_ are investigated by using DFT calculations for seven dopants (F, Cl, S, N, P, SO_4_, and PO_4_). We found (1) S, N and P doping remarkably reduces its band gap from 4.82 eV to 3.86 eV, 3.79 eV and 2.69 eV, respectively; (2) the band gap reduction is induced by the electron transfer to the dopant atoms; (3) F, Cl, SO_4_, and PO_4_ doping shifts its valence band to be lower than the oxidation potential of O_2_/H_2_O to render its band structure appropriate for photocatalytic water splitting. These results suggest that not only electrical conductivity of 2D Mg(OH)_2_ can be increased but also their band structure be aligned by using the proposed anion doping strategy. These results enable a new photocatalytic materials design approach while offering exciting possibilities in applications of high-current electrolysis, chemical gas sensing, and photocatalysis.

## 1. Introduction

Widely available and non-toxic Mg(OH)_2_ represents a fine example of multifunctional compounds with extensive technological and industrial applications [1], such as removing pollutants using its adsorptive and coagulative properties [2,3,4,5,6], acting as an effective antibacterial agent [7], protecting paper by reducing the paper ageing [8], adding as a component in organic-inorganic composite membrane [9,10], utilizing as a new-generation flame retardant and smoke suppression [11,12]. In addition, Mg(OH)_2_ has been considered as a photocatalyst for degradation of organic dyes in wastewaters, such as Rhodamine B [13], and methyl orange [14], and for CO_2_ conversion to solar fuels such as CO, CH_4_, CH_3_OH, HCOOH, and HCOH [15,16]. Owing to different deposition and processing methods as well as characterization tools, Mg(OH)_2_ reports a scattered experimental band gap values from 5.17 eV [17,18], 5.47 eV [19], 5.70 eV [17], to 7.60 eV [20], and is considered a wide gap insulator. Accordingly, it is used as a buffer layer in heterostructure solar cells [18,21] and to suppress recombination of photogenerated electrons in dye-sensitized solar cells [22,23]. However, the low electric conductivity of Mg(OH)_2_ has proven a challenge in important applications, such as high-current electrolysis, chemical gas sensing and photocatalysis.

Efforts have been made to adjust the electronic structure of Mg(OH)_2_ mainly by cation doping to exploit its photocatalytic applications. Cu doping can render Mg(OH)_2_ n-type or p-type conductive since Cu atoms can be a shallow donor at the interlayer site or a deep acceptor at the substitutional site replacing Mg [24,25]. Subsequently, a p-n homojunction is fabricated using Cu-doped Mg(OH)_2_ [26]. Cobalt doping is examined to tune the bandgap of Mg(OH)_2_, and 10% Co-doping slightly narrows the band gap from 5.47 eV of pure Mg(OH)_2_ to 5.26 eV [19]. Doping of Na, K, Cu, Ag, F, Cl, and C at interlayer sites, and K, Ca, Mn, Fe, Co, Ni, Cu, Zn, Al, Si, Sn, and C at substitutional sites were evaluated to give Mg(OH)_2_ n-type or p-type conductivity [27]. However, remarkable bandgap variation was not observed. It is encouraging to notice that the heavily C-doped Mg(OH)_2_ films are electrically conducting [28,29]. It is initially speculated that C atoms replace H and bond to O [20], but isolated C atoms cannot induce significant conduction [27]. C atoms of large content are supposed to form a 2D graphite-like structure in Mg(OH)_2_ to render it metallic conductive. There is currently no guideline to direct toward dopant selection to tune the electronic structure of Mg(OH)_2_.

Recently, 2D Mg(OH)_2_ was successfully synthesized by mechanical exfoliation from its layered crystals [30]. The bandgap for 2D Mg(OH)_2_ is 4.80 eV from HSE06, being of insulating nature with wide bandgap [30]. It has been reported to be a decent layered material to construct heterostructures. Owing to the staggered interfacial bandgap and the electric field tunable electronic structures, WS_2_/Mg(OH)_2_ [31] and graphene/Mg(OH)_2_ [32] heterostructures can be an important candidate for various optoelectronic device applications in nanoscale. The potential application of Cs_3_Sb/Mg(OH)_2_ heterostructure for high-performance photocathodes [33], of *h*-AlN/Mg(OH)_2_ heterostructure for the light harvesting [34], and of *h*-BN/Mg(OH)_2_ heterostructure for magnetic tunnel junction memory devices [35] are theoretically explored. Equipped with decent band edge positions and excellent optical absorption property near the visible-light region, the van der Waals vertical heterostructures constructed by blue phosphorus/Mg(OH) [36], *g*-GaN/Mg(OH)_2_ [37], ZnO/Mg(OH)_2_ [38], MoS_2_/Mg(OH)_2_ and WS_2_/ Mg(OH)_2_ [39] can act as a promising photocatalyst in hydrogen evolution and oxygen evolution reactions for water splitting. Charge-transfer and ionic interactions may play additional substantial role in holding these heterostructures together. These heterostructures possess intrinsic type-II band alignment. Moreover, the photogenerated charges can be effectively separated by a large built-in electric field across the interface. Unfortunately, there is no report on single phase 2D Mg(OH)_2_ being applied in these fields.

Therefore, we aim to engineer the electronic structure of single phase 2D Mg(OH)_2_ to function like the above-mentioned heterostructures. Since the valance band top and conduction band bottom of Mg(OH)_2_ are predominantly comprised of O-2p bonding and anti-bonding states [40], it suggests that anion doping may exhibit appreciable influence on the electronic structure of Mg(OH)_2_. Reports of successful engineering of its bandgap via anion doping are so far a rarity. Particularly, our interest in extending its applications in water splitting requires a quantitative understanding of the absolute band alignment of the anion doped Mg(OH)_2_, which are also unavailable in literature. Therefore, in this report we carry out theoretical investigations of electronic properties of Mg(OH)_2_ with a series of anion dopants via density-functional theory (DFT), with the objective to find new physics and obtain doping rule for the anion doping effect. This work elucidates the variation of doping energetics and energy bandgap of 2D Mg(OH)_2_, with emphasis on (1) disclosing crucial factors for bandgap narrowing, and (2) ascertaining the absolute band alignment for photocatalytic applications. It is expected that, by systematically screening dopant candidates, this work will guide and speed up experimentation to engineer Mg(OH)_2_ electronic structures for photocatalytic applications.

## 2. Methods

The first-principles calculations were conducted using a periodic supercell model employing the Vienna Ab-initio Simulation Package (VASP) [41] with the Perdew-Burke-Ernzerhof (PBE) generalized gradient approximation (GGA) exchange-correlation functional [42]. A projector augmented wave (PAW) method [43,44] was used as a plane wave basis set. For the plane-wave expansion, a 500 eV kinetic energy cutoff was set according to the cutoff energies testing with the energy error of 0.001 eV. The contribution of long range dispersion (van der Waals interaction) based on the DFT+D3 correction method [45] was applied to all calculations. The dipole corrections were also applied. A 4×4 expansion of Mg(OH)_2_ unit cell of ab plane was employed to generate the 2D Mg(OH)_2_ system with 80 atoms. HF, HCl, H2S, NH_3_, PH_3_, H_2_SO_4_ and H_3_PO_4_ are selected to generate dopant via dehydration reaction with the supercell, which gives a dopant concentration of about 3%. These reactions are equivalent to substitute one –OH in the supercells with –F, –Cl, –HS, –NH_2_, –PH_2_, –HSO_4_ and –H_2_PO_4_, respectively. These dopants are denoted as F, Cl, S, N, P, SO_4_ and PO_4_ for simplicity. At least 20 Å vacuum is placed on both sides of the 2D Mg(OH)_2_ system to avoid images interaction in the periodic boundary condition. To calculate the total energies of isolated molecules, the simple cubic supercell with a lattice constant of 30 Å was employed.

The convergence criteria for the geometric optimization and energy calculation were set as follows: (1) self-consistent field energy tolerance is 1.0 × 10^−5^ eV, (2) all the atoms in the systems were fully relaxed and maximum force tolerance on each atom is smaller than 0.01 eV/Å. A Monkhorst−Pack K-points mesh [46] was used for sampling the Brillouin zone, where the number of K-points (N_K_) is changed to keep (N_K_ × L) with L being the lattice constant equal to ~45 Å and ~75 Å for structural relaxations and electronic calculations, respectively. The Heyd–Scuseria–Ernzerhof (HSE06) hybrid functional [47] was also used for electronic structure calculations for all systems optimized with PBE functional. We used the following standard parameters for the mixing parameter α and the adjustable parameter ω controlling the short-range interaction: α = 0.25 and ω = 0.20, which work well in terms of correcting the band gaps of common semiconductors [48]. The smearing value was set as 0.1 eV. The charge in an atom was defined as the difference between the valence charge and the Bader charge. The Bader charge was determined with the Bader scheme of charge density decomposition [49,50].

## 3. Results and Discussion

### 3.1. Electronic Structure of Bulk and 2D Mg(OH)_2_

We first calculated the structural parameters of bulk and 2D Mg(OH)_2_ using PBE functional. The optimized and experimental lattice constants of Mg(OH)_2_ crystal, which has a trigonal structure of space group $P\bar{3}m1$ (164), were presented in Table 1. The calculated structural parameters show good consistence with the experimental values [51], with an average error of about 2%. Our calculations also demonstrate excellent agreement with the reported first-principle calculations [30,36].

**Figure 1:**
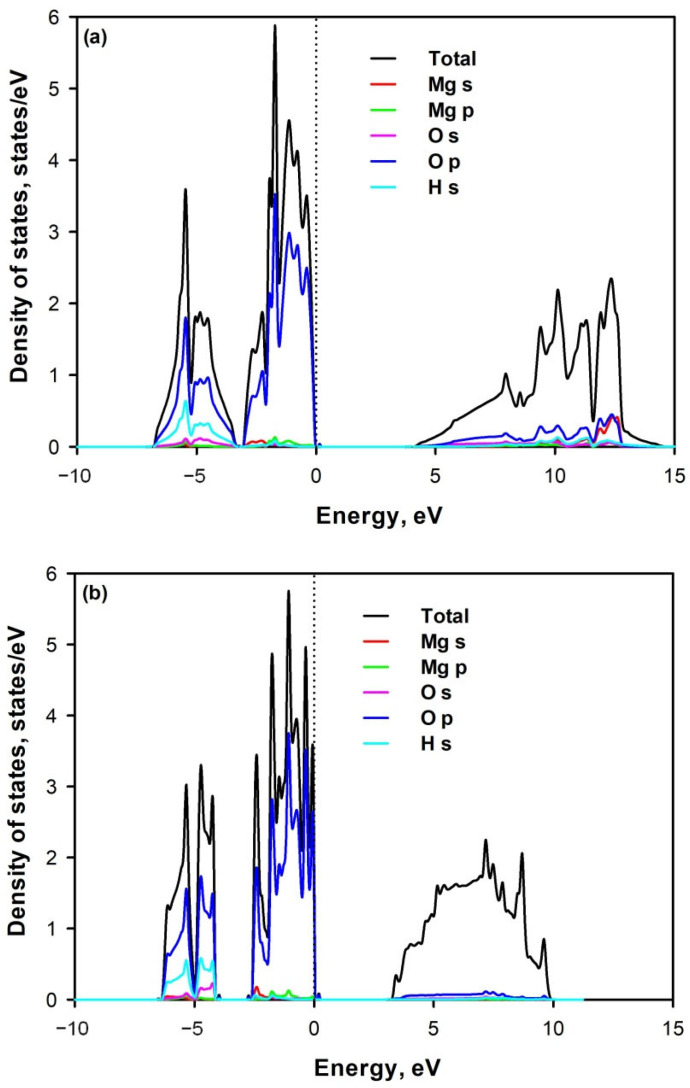
Total and partial density of states (PBE) of (**a**) bulk and (**b**) 2D Mg(OH)_2_. The Fermi level is set as zero energy.

The electronic structures of bulk and 2D Mg(OH)_2_ are shown in Figure 1. Both the bulk and 2D Mg(OH)_2_ indicate the characteristic of the semiconductor with a wide bandgap of 4.424 eV and 3.357 eV as shown in Table 2, respectively. Bulk Mg(OH)_2_ has larger band gap than 2D Mg(OH)_2_. The calculated values are smaller than the scattered experimental values, since DFT calculation based on GGA generally underestimates the bandgap due to lack of proper description of functionals used in calculations [52]. No significant discrepancy of contribution by each type of atom to different regions of the electronic bands is observed between bulk and 2D Mg(OH)_2_. It can be seen that the valence bond maximum (VBM) is predominantly contributed by O-2p orbitals, and the conduction bond minimum (CBM) is comprised of major O-2p orbitals and minor H-1s orbitals. The valence bands consist of the upper part (−4–0 eV) and the lower part (−6–−4 eV). The upper part is mainly contributed by the O-2p orbitals, and the lower part is contributed by the O-2p orbitals and H-1s orbitals. There are minor contributions of H-1s orbital and Mg-2s orbitals to the conduction bonds. The conduction band appears as a well-delocalized state. This supports the claim that the outermost oxygen electrons predominately affect the total DOS [53]. This agrees with the observations that cation doping cannot effectively tune its electronic structure.

Hybrid HSE06 functional was further employed to calculate the bandgaps of bulk and 2D Mg(OH)_2_ with the results listed in Table 2. By combining the short-range Fock exchange and semi-local long-range exchange, it gives more accurate bandgap values. The calculated bandgap value for the bulk Mg(OH)_2_ is comparable with the experimental values. These results are also in great agreement with previous theoretical calculations [30,31].

### 3.2. Doping Energy

The chemical reaction involved in doping can be expressed as dehydration process as follows,

(1) $${\mathrm{Mg}}_{n}{\left(\mathrm{OH}\right)}_{2n}+\mathrm{HX}\to {\mathrm{Mg}}_{n}{\left(\mathrm{OH}\right)}_{2n-1}X+H_{2}O,$$

where ${\mathrm{Mg}}_{n}{\left(\mathrm{OH}\right)}_{2n}$ and ${\mathrm{Mg}}_{n}{\left(\mathrm{OH}\right)}_{2n-1}X$ are pristine and doped 2D Mg(OH)_2_ supercells, respectively; while HX and $H_{2}O$ are the molecule used for doping and released water molecule, respectively. The doping energy E_d_ is defined as the total energy change in pristine and doped 2D Mg(OH)_2_ supercells and isolated molecules obtained using the DFT calculations as follows,

(2) $$E_{d}=E\left({\mathrm{Mg}}_{n}{\left(\mathrm{OH}\right)}_{2n-1}X\right)+E\left(H_{2}O\right)-E\left({\mathrm{Mg}}_{n}{\left(\mathrm{OH}\right)}_{2n}\right)-E\left(\mathrm{HX}\right),$$

(3) ΔG=ΔH−TΔS=ΔE+PΔV−TΔS,

The change in Gibbs free energy of the above doping reaction ΔG is calculated using Equation (3), where ΔH is the enthalpic energy change, and ΔE the internal energy change. Since the lattice parameters of the supercells are fixed in our doping calculations, the volume changes (ΔV) are not taken into consideration, therefore, the PΔV work term can be ignored. Moreover, we assume that the entropic contribution (TΔS) with the order of $k_{B}T$ at room temperature could be negligibly small compared to the internal energy difference with the order of few eV. Therefore, ΔG can be further approximated to ΔE, which is equal to E_d_ according to the definition. Consequently, E_d_ can be regarded as the change of Gibbs free energy of the reaction to generate the dopants in the supercell. A negative E_d_ value indicates that the doping reaction is a spontaneous process, while a positive E_d_ value indicates that the doped system is in a metastable state at the local minima, and thus the doped system is not as thermodynamically stable as the pristine 2D Mg(OH)2.

The calculated doping energy values for various dopants are listed in Table 3. It can be seen that F, Cl, SO_4_ and PO_4_ doping reaction are exothermic, while S, N and P doping reaction are endothermic. Considering that 2D Mg(OH)_2_ can be regarded as a weak base, since both NH_3_ and PH_3_ are weak bases, therefore, the doping energy for N and P doping reaction is positive. As nitrogen has a smaller size and is more electronegative than phosphorous, the Mg-P bond length is larger than the Mg-N bond length. The weak covalent Mg-P bond leads to the highest doping energy among the studied dopants. However, it is unexpected that the reaction energy of H_2_S with 2D Mg(OH)_2_ supercell is positive, as H_2_S is a weak acid. Its E_d_ value is much smaller than those for N and P doping. This may be due to the large size of S. The Mg-S bond length is a slight shorter than that of Mg-P bond. The stronger acid HF, HCl, H_2_SO_4_ and H_3_PO_4_ demonstrate negative doping energy, implying that their reactions with 2D Mg(OH)_2_ can spontaneously occur.

**Figure 2:**
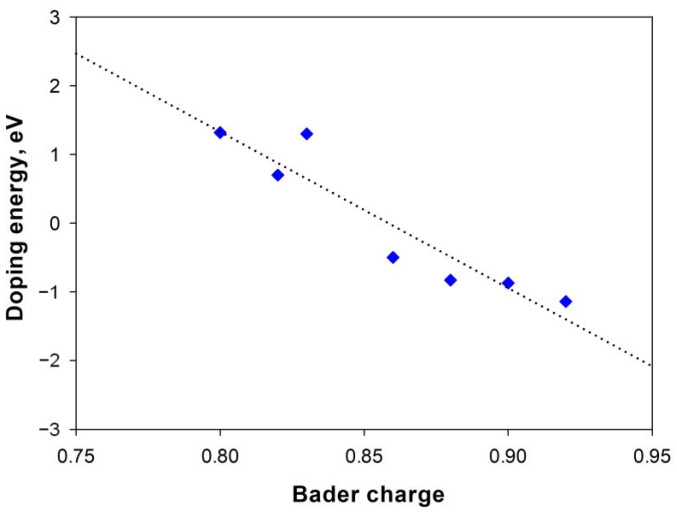
Variation of doping energy with Bader charge for various dopants. The dotted trend line is a guide to the eye.

Table 3 also presents the Bader charge analysis results together with the Mg-dopant bond length. All dopants accept different amount of electrons donated by Mg atoms. A linear relation is observed between the accepted Bader charge of dopants and their doping energy in Figure 2. The larger Bader electrons are transferred from Mg to dopant, the more negative doping energy for the doping reaction, the easier for the doping reaction to take place. It seems that a large Bader charge transfer facilitates the formation of strong covalent bonding between Mg and dopant. Therefore, it is beneficial to the doping reaction and gives rise to the stable doped 2D Mg(OH)_2_. The calculated doping energy follows the order H_2_SO_4_ > H_3_PO_4_ > HF > HCl. This is not in agreement with their acid strength in water H_2_SO_4_ > HCl > H_3_PO_4_ > HF, which may be due to the lack of solvation effect in our calculations. On the other hand, the Mg-dopant bond length appears to be irrelevant to both the doping energy and Bader charge accepted by the dopant, as it is mainly affected by the dopant size. This suggests that dopant size could not be used as a reliable parameter for screening out dopant candidates to tailor the Mg(OH)_2_ electronic structure.

**Figure 3:**
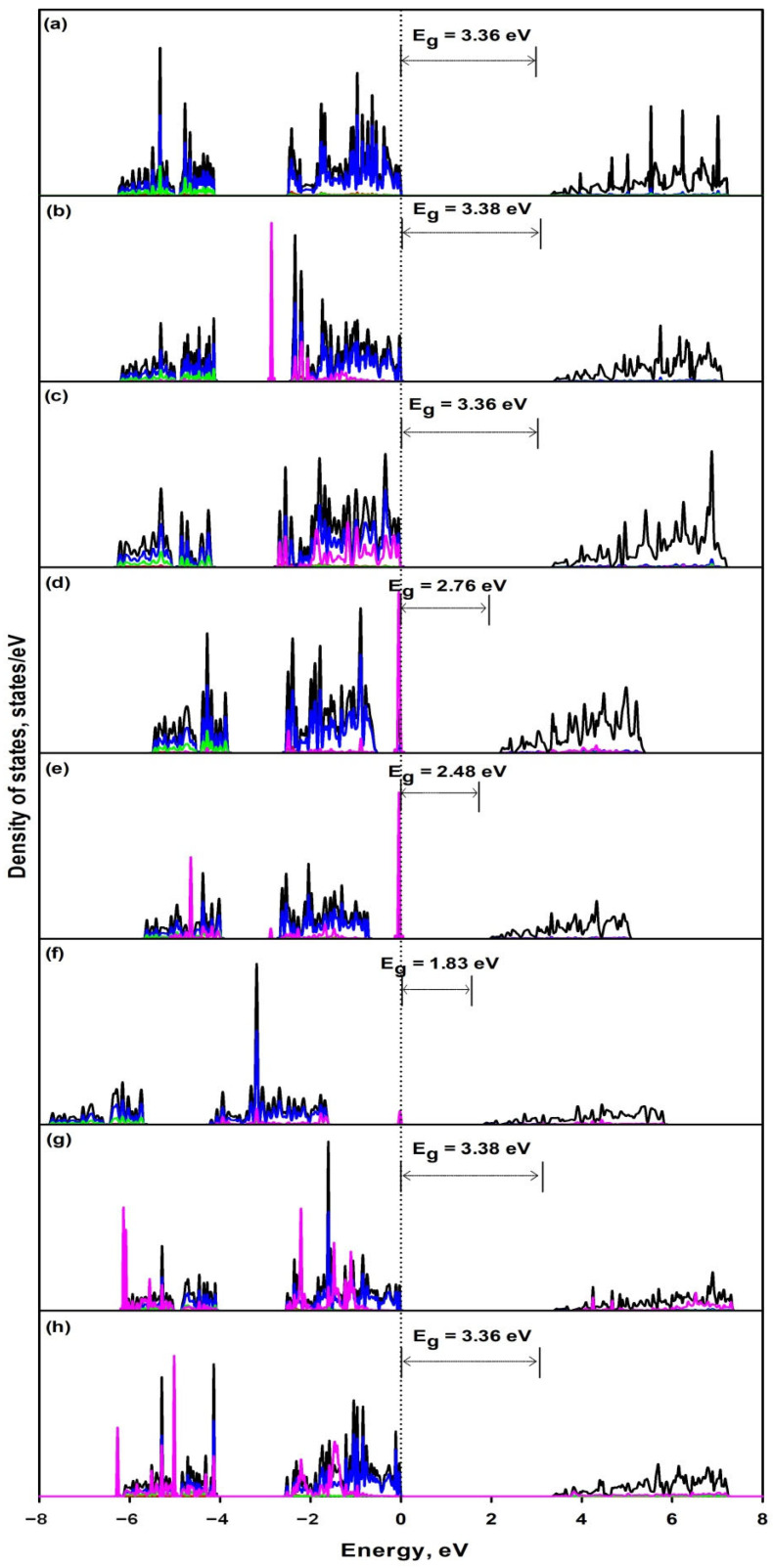
Total and partial DOS (PBE) of various 2D Mg(OH)_2_ including (**a**) pristine (**b**) F, (**c**) Cl, (**d**) S, (**e**) N, (**f**) P, (**g**) SO_4_, (**h**) PO_4_. The Fermi level is set as zero energy. The black line stands for TDOS, while the red, blue, green and pink lines stand for contribution of Mg atoms, O atoms, H atoms and dopant, respectively.

Figure 3 shows the total and partial DOS of doped 2D Mg(OH)_2_ in comparison with pristine 2D Mg(OH)_2_. It can be seen that different dopants contribute to different valence band regions and make slight contributions to the conduction band. It is observed that F and Cl dopants make substantial contributions to upper part of valence bands while SO_4_ and PO_4_ dopants make significant contribution to both upper and lower valence bands, however, they make no contributions to the valence band top. Therefore, they have negligible effect on the bandgap energy as seen in Table 4. On the other hand, S, N and P dopants form an impurity state (a free-electron-like state) in the bandgap, effectively reducing the bandgap energy. The shift of the bandgap energy to UV/visible light region will increase the visible light absorption, and reduce the energy required for electron transfer from valence band to conduction band. This will substantially increase the density of the excited-state electrons in the conduction band, and is beneficial to enhanced electrical conductivity. However, the impurity states in the bandgap may act as recombination centers, and reduce the photocatalytic efficiency. Table 4 also presents the more accurate energy bandgap calculated by HSE06 functionals. S, N, and P doping has significantly reduced the bandgap from 4.82 eV to 3.86 eV, 3.79 eV, and 2.69 eV, respectively. Besides the band gap, the work function is another critical factor to determine the photocatalytic performance. The behavior of the electron and hole formed by incident light is strongly affected by the work function. For example, the low work function is conducive to the migration of excited electrons from the conduction band to the crystal surface of Cu_2_O for significantly increased photocatalytic activity [54]. The work function (E_WF_) is essentially the Fermi level referenced to the vacuum level ($E_{\mathrm{WF}}=E_{\mathrm{vac}}-E_{F}$, where E_F_ denotes the Fermi energy level, and E_vac_ is the vacuum energy). It is controlled by the atomic interaction, which mainly reflects the electron behavior of material surface. The Bader charge analysis indicates that the O atom of pristine 2D Mg(OH)_2_ has the largest accepted Bader charge of being +1.37, which is much larger than those of the anion dopants ranging from +0.80 to +0.92 as shown in Table 3. This implicates that the surface H atoms in the anion-doped 2D Mg(OH)_2_ demonstrate higher collection of electrons, which will bring the Fermi energy closer to the conduction band, an increase of E_F_ value [55]. This might bring about the reduction of work function in the anion doped 2D Mg(OH)_2_ if the Evac value is not substantially affected by the doping. The work function is remarkably reduced from 4.44 eV to 4.24 eV and 3.85 eV for N and P doping, respectively.

**Figure 4:**
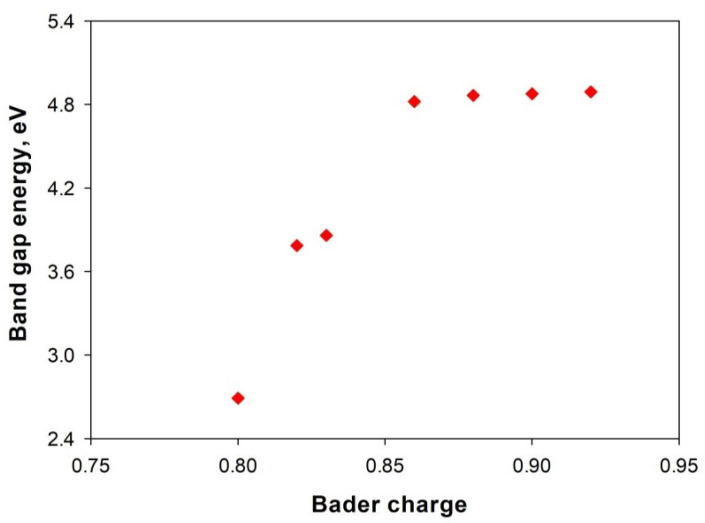
Bandgap energy of doped 2D Mg(OH)_2_ with Bader charge of dopant.

The dependence of bandgap energy of doped 2D Mg(OH)_2_ on the Bader charge by the dopant is shown in Figure 4. It is observed that a decrease of the accepted electron by the dopant narrows the bandgap energy. This indicates that the valence band top appears to be determined by the accepted electron amount of the dopant. The bandgap is narrowed with a reduction of accepted electron by the dopant. Electronegativity stands out to be a candidate screening property since it quantifies the tendency of an atom or a functional group to attract electrons to it. The decrease of electronegativity of the dopant is consistent with the bandgap narrowing. When the accepted Bader charge by the dopant reaches 0.86, the bandgap energy remains nearly unchanged, and a further increase of the accepted Bader charge by the dopant marginally affects the bandgap. This makes it possible to tailor the accepted electrons by choosing the dopants with specified electronegativity to engineer the bandgap energy. This also supports our claim that cation doping has little effect on altering the bandgap.

**Figure 5:**
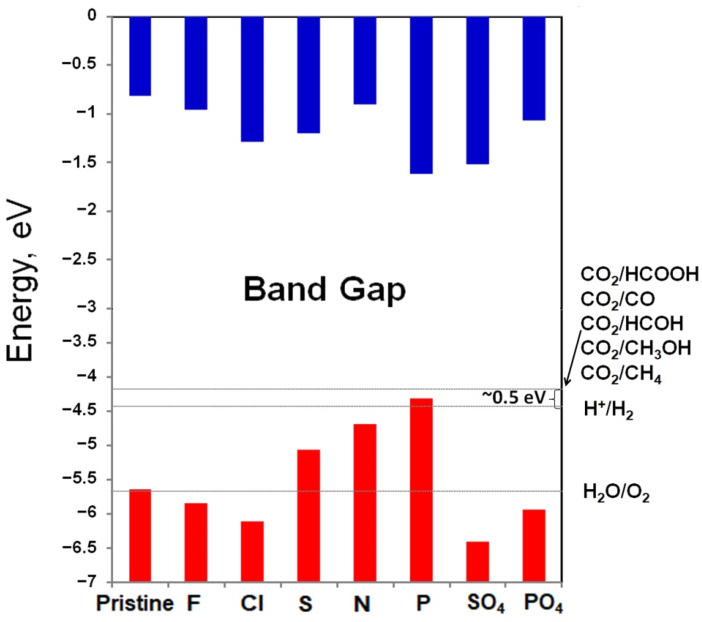
The absolute band alignment of various 2D Mg(OH)_2_ calculated by HSE06. The vacuum level is set as zero energy. Blue and red colors stand for conduction band and valence band, respectively.

To render the 2D Mg(OH)_2_ potential photocatalytic for water splitting, it must have decent band-edge positions for the redox potentials, where the CBM energy is higher than the reduction potential (−4.44 eV) of H^+^/H_2_, while the VBM energy is lower than the oxidation potential (−5.67 eV) of O_2_/H_2_O. When energy losses due to overpotential are counted, a much larger bandgap is usually needed for appreciable water splitting reaction [56]. For the photocatalytic reduction of CO_2_, the required potential for CO_2_ reduction to CH_3_OH is even higher (−0.38 V in comparison with 0 V of water at PH 7) [57]. Therefore, the bandgap energy for an excellent semiconductor photocatalyst is $E_{g}<3.1\;\mathrm{eV}$ for visible light response [58]. Figure 5 shows the absolute band alignment of doped 2D Mg(OH)_2_ in comparison with pristine 2D Mg(OH)_2_. It is seen that the CBM of all 2D Mg(OH)_2_ are higher than the reduction potential of H^+^/H_2_, and also higher than the reduction potential of CO_2_, indicating that they are capable of the reduction reaction. Figure 5 indicates that VBM of pristine 2D Mg(OH)_2_ is slightly larger than the oxidation potential of O_2_/H_2_O. This supports our observation that there is no direct application of 2D Mg(OH)_2_ in photocatalytic water splitting without constructing vertical heterostructures with additional layered materials. VBM of F, Cl, SO_4_ and PO_4_ doped 2D Mg(OH)_2_ moves downwards to lower energy due to the dopant’s contribution to the valence bands, and becomes lower than the oxidation potential of O_2_/H_2_O. This makes the doped 2D Mg(OH)_2_ feasible to be applied for photocatalytic water splitting. Their bandgap is adversely higher than 3.1 eV for visible light response, with their bandgap being close to 4.80 eV of pristine 2D Mg(OH)_2_. Though it is encouraging that S, N, and P doping remarkably reduces the bandgap of 2D Mg(OH)_2_, its VBM is moved upward to be over the oxidation potential of O_2_/H_2_O. These doped 2D Mg(OH)_2_ cannot stand out as attractive candidates for photocatalytic water splitting applications. It is expected that S, N and P doping 2D Mg(OH)_2_ will substantially improve the efficiencies of photocatalytic degradation of organic dyes with the remarkably reduced bandgap and work function. It can even be inferred that a coupling doping of these anions may be a promising approach to achieve desirable bandgap and band alignment for photocatalytic water splitting. Therefore, our results establish a systematical methodology for the investigation of additional anion doping.

Great efforts have been devoted to developing effective and earth-abundant non-noble photocatalysts for water splitting to produce clean and eco-friendly hydrogen. Among all the methods, doping anions or cations stands out as an attractive approach to modify electronic structure of the catalysts. Particularly, anion doping is expected to more effectively modulate electronic structure, and thus improving photocatalytic performance. Anion doping into the oxygen site has emerged as a new strategy for tuning the chemical and physical properties of metal oxides, and thus for regulating their catalytic behavior [59]. F-, Cl- and Br- doping has been applied to tune the electronic structure of perovskite oxides since partial occupation of non-oxide anions at the oxygen sites could introduce many interesting properties [60]. Br- and Cl- co-doping causes the absorption edge of TiO_2_ to shift to a lower energy region and demonstrates improved photocatalytic activity for water splitting into H_2_ and O_2_ under ultraviolet light [61]. Doping with carbon, nitrogen, and sulfur, yields promising second-generation photocatalysis with TiO_2_ [62]. Anion regulation is regarded as an emerging but effective method to improve the catalytic activity of transition metal compounds [63]. Our results shed greater light on the positive influence of anion-doping to boost its applications to 2D layered materials.

## 4. Conclusions

Doping is a useful tool for the modulation of the electronic structure of semiconductors. Since the bandgap region of the electronic structure of 2D Mg(OH)_2_ is predominantly controlled by the O-2p electrons, anion doping may make direct contribution to the valence band top or conduction band bottom depending on their electronegativity, which will explicitly affect the bandgap and the band alignment. On the other hand, cation doping will interact with O atoms to form bonding of different strength to implicitly adjust the valence band top or conduction band bottom. Therefore, anion doping could be more effective in engineering the electronic structure of 2D Mg(OH)_2_. Consequently, a systematic anion doping strategy, by using F, Cl, S, N, P, SO_4_ and PO_4_, is demonstrated to engineer the band structure for general photocatalytic applications. Our first-principles calculations indicate that F, Cl, SO_4_ and PO_4_ doping is energetically favored while S, N and P doping becomes endothermic. The accepted electron by the dopant modulates the bandgap energy of 2D Mg(OH)_2_. S, N and P doping remarkably reduces the bandgap energy from 4.82 eV to 3.86 eV, 3.79 eV and 2.69 eV, respectively; while F, Cl, SO_4_ and PO_4_ doping shows a slight alteration of the bandgap. The absolute band alignment in term of H_2_O and CO_2_ redox potentials discloses that F, Cl, SO_4_, and PO_4_ doped 2D Mg(OH)_2_ has appropriate band structures. Therefore, doping of these anions may achieve desirable bandgap and band alignment for photocatalytic water splitting. These results enable a new photocatalytic material design approach while offering exciting possibilities in applications of high-current electrolysis, chemical gas sensing, and photocatalysis.

## Figures and Tables

**Table 1:** Lattice parameters of bulk and 2D Mg(OH)_2_.

Structure	Calculated			Experimental [51]		
	a	b	c	a	b	c
Bulk	3.19	3.19	4.88	3.15	3.15	4.77
2D	3.18	3.18	-			

**Table 2:** Calculated bandgap (E_g_) and work function (E_WF_) of bulk and 2D Mg(OH)_2_.

	E_g_ (PBE) (eV)	E_g_ (HSE06) (eV)	E_WF_ (PBE) (eV)	E_WF_ (HSE06) (eV)
Bulk	4.22	7.30	4.43	6.35
2D	3.36	4.82	4.44	5.54

**Table 3:** Doping energy (E_d_) of each dopant and its Bader charge and bond length with nearest Mg in doped 2D Mg(OH)_2_.

Dopant	E_d_ (eV)	Bader Charge	Mg-Dopant Bond Length (Å)
F	−0.83	+0.88	2.09
Cl	−0.50	+0.86	2.65
S	0.70	+0.82	2.72
N	1.30	+0.83	2.18
P	1.32	+0.80	2.92
SO_4_	−1.14	+0.92	2.40
PO_4_	−0.87	+0.90	2.321

**Table 4:** Calculated bandgap (E_g_) and work function (E_WF_) of doped 2D Mg(OH)_2_.

Dopant	E_g_ (PBE) (eV)	E_g_ (HSE06) (eV)	E_WF_ (PBE) (eV)	E_WF_ (HSE06) (eV)
F	3.38	4.89	4.50	5.19
Cl	3.36	4.82	4.96	5.66
S	2.76	3.86	4.29	4.60
N	2.48	3.79	3.69	4.24
P	1.83	2.69	3.32	3.85
SO_4_	3.38	4.88	5.16	5.94
PO_4_	3.36	4.87	4.757	5.48

## Data Availability

The data presented in this study are available upon request from the corresponding author.
